# Initial Costs of Ebola Treatment Centers in the United States

**DOI:** 10.3201/eid2202.151431

**Published:** 2016-02

**Authors:** Jocelyn J. Herstein, Paul D. Biddinger, Colleen S. Kraft, Lisa Saiman, Shawn G. Gibbs, Philip W. Smith, Angela L. Hewlett, John J. Lowe

**Affiliations:** University of Nebraska Medical Center College of Public Health, Omaha, Nebraska, USA (J.J. Herstein, J.J. Lowe);; Harvard Medical School, Boston, Massachusetts, USA (P.D. Biddinger);; Emory University, Atlanta, Georgia, USA (C.S. Kraft);; Columbia University Medical Center, New York, New York, USA (L. Saiman);; Indiana University School of Public Health, Bloomington, Indiana, USA (S.G. Gibbs);; University of Nebraska Medical Center College of Medicine, Omaha (P.W. Smith, A.L. Hewlett)

**To the Editor:** The 2014–2015 outbreak of Ebola virus disease (EVD) in West Africa was unprecedented in scale and scope. During the outbreak, 11 patients with EVD were cared for in the United States (*1*). Safely caring for patients with suspected EVD requires specialized protocols and training for hospital staff in the use of personal protective equipment (PPE) and isolation precautions (*2*,*3*). The care of a hospitalized patient with confirmed EVD in high-level isolation units requires large specialized teams of nurses, physicians, laboratory technologists, environmental service workers, and waste management specialists, and inpatient care may continue for weeks (*3*,*4*). The staff-to-patient ratio necessary to care for a patient with EVD in high-level isolation is much higher than that in a typical intensive care unit because of the extensive PPE used and the need for partners to assist with PPE donning and doffing.

In response to preparedness challenges in the United States, the Centers for Disease Control and Prevention recommended a multitiered framework of hospitals with advanced capabilities for Ebola care: frontline facilities, Ebola assessment hospitals, and Ebola treatment centers (ETCs) (*2*). Within this federal framework, 55 hospitals in the United States have been designated by their states as ETCs, which have the advanced capabilities required to provide medical care to patients with confirmed EVD throughout their illness (*5*). Although the cost of preparing these healthcare facilities to care for EVD patients was believed to be substantial (*5*–*7*), we aimed to directly survey the ETCs to determine the costs incurred to prepare their facilities to manage and treat EVD patients.

In April 2015, we sent a 19-question electronic survey to all 55 ETCs, including the 3 preexisting biocontainment patient care units (Technical Appendix). Participation was voluntary, and individual responses were confidential. The survey assessed the ETCs’ general organization and the costs incurred to establish the ETC. Of the ETCs, 45 indicated interest in participating in the establishment of the United States Highly Infectious Diseases Network to establish infection control metrics and competencies for high-level patient isolation centers. The Institutional Review Board of the University of Nebraska Medical Center declared this study exempt.

Of the 55 ETCs, 47 (85.5%) responded to the survey; 45/47 reported the total costs incurred to establish their ETC, and 43/47 provided a detailed assessment of costs. The 45 ETCs reporting total costs incurred a cumulative total of $53,909,701 (mean $1,197,993/ETC) to establish the ETCs (Table). The most costly activity was facility construction and modifications. Costs incurred to provide initial training for staff averaged $267,075 (range $10,000–$1,624,639). Each ETC spent $172,581 (mean per facility; range $3,000–$560,000) on other expenses not included in the 5 specified categories (Table). Examples of additional costs included computer hardware and software, nonmedical equipment, office supplies, and employee apparel. Costs and expenses allocated to specific purchases varied by region (Technical Appendix Figures 1, 2).

**Table:** Initial costs in US$ incurred by 45 Ebola treatment centers in the United States*

Cost scale	Total costs	Construction/ facility modifications	PPE supplies	Staff training	Unit planning	Laboratory equipment	Non-PPE and nonlaboratory supplies and equipment
Average	1,197,993	420,502	213,347	267,075	176,713	99,106	172,581
Median	1,000,000	202,980	110,000	150,000	82,000	84,000	100,000
High	6,556,457	3,839,000	1,067,573	1,624,639	1,200,000	317,406	560,000
Low	51,500	8,500	10,000	10,000	15,000	0	3,000
Sums†	53,909,701	16,820,080	8,747,240	10,950,072	4,947,966	3,865,124	6,385,513

*PPE, personal protective equipment. †Summarized data were collected through self-report by individual treatment centers through an electronically administered survey.

With the exception of 3 hospitals that had preexisting biocontainment units, 52 hospitals had to undertake novel activities to prepare to care for patients with EVD, including development of plans, recruitment of facility leadership, recruitment and training of a multidisciplinary team of volunteers, and purchase of specialized supplies and equipment. The nearly $54 million in previously unbudgeted expenses was a substantial financial burden on the ETCs. Wide variations for overall expenditures and for specific types of expenditures were noted.

Because 10 ETCs did not report financial data, the overall costs reported here do not fully estimate the expenses incurred by ETCs. Furthermore, these overall costs represent only the initial start-up costs of establishing ETCs and do not include the costs of ongoing maintenance such as resupplying validation reagents for the laboratory, purchasing supplies and equipment, continual training of staff, or testing the units and programs.

This study had limitations. We could not validate self-reported data from the ETCs with information from expense reports. We also acknowledge that many additional hospitals undertook similar efforts to those of the designated ETCs but were not included in this survey (*8*). The costs incurred by public and private public health organizations also were not included.

In conclusion, we have described the initial preparation costs incurred by designated ETCs in the United States. The substantial start-up costs as well as ongoing maintenance costs of EVD programs underscore the need for specialized facilities to treat EVD (*9*,*10*). A tiered nationwide network of healthcare facilities that can rapidly identify, isolate, and treat patients with EVD has been established to improve the nation’s preparedness for EVD and can serve as a valuable resource for future outbreaks of other highly infectious diseases. Ongoing resources will be needed to sustain the readiness of such a network.

Technical Appendix Questionnaire sent to all Ebola virus disease treatment centers in the United States, average total costs incurred in each of the 10 US Health and Human Services regions (US$), and the interquartile ranges of the distribution of costs of 45 Ebola treatment centers.
