# Supplementary material for: Initial Costs of Ebola Treatment Centers in the United States
Source: Emerg Infect Dis. 2016 Feb;22(2):350–2. doi: 10.3201/eid2202.151431 (PMC4734525; doi:10.3201/eid2202.151431)
Supplement: Technical Appendix — Questionnaire sent to all Ebola virus disease treatment centers in the United States, average total costs incurred in each of the 10 US Health and Human Services regions (US$), and the interquartile ranges of the distribution of costs of 45 Ebola treatment centers. [file 15-1431-Techapp-s1.pdf]

Article DOI: http://dx.doi.org/10.3201/eid2202.151431

Initial Costs of Ebola Treatment Centers in the United States

Technical Appendix

A.1 General Aspects. The facility addressed in this checklist:

A.1.a) Please indicate the name of the EVD/Special Pathogens Care Treatment sponsoring hospital and location:

Hospital: ______________________________ City/State: ________________

A.1.b) Is the hospital applying to be the regional center? ☐YES ☐NO

A.1.c) EVD inpatient care facility is located within:

i) Main Hospital Building(s) ☐YES ☐NO

If yes: Located within

☐Academic/teaching hospital

☐Referral / regional hospital (but not Academic Medical Center)

☐Other (Armed Forces/Infectious Disease Center): ______________________________

ii) Independent facility (stand alone facility) ☐YES ☐NO

If yes, is facility located on the same campus as main hospital building(s)? ☐YES ☐NO

No information / other (please specify):

____

A.2. High level isolation Capacity:

A.2.a) Number of Ebola or Highly Infectious Disease ISOLATION ROOMS AND BEDS

i) Maximum number of high level patient isolation rooms and beds that can be used simultaneously

number of rooms: ________________________ number of beds (total): __________________

ii) Bed capacity for adult patients n = ________________________

Critical care capable? ☐YES ☐NO

iii) Bed capacity for pediatric patients n = ________________________

Critical care capable? ☐YES ☐NO

No information / other (please specify):

____

A.3. Location of isolation rooms

A.3.a) Where are the isolation rooms specifically located?

i) In a separate ward, but within the same building as other main hospital facilities ☐YES ☐NO

If yes, is the air handling for the ward separate from the air handling for the rest of the building?

☐YES ☐NO

ii) In separate rooms, but in the same ward as other hospital facilities ☐YES ☐NO

(e.g. Inf. Diseases Ward, or ICU)

If yes, is there a physical barrier (wall or other) separating the isolation rooms from the rest of the ward? ☐YES ☐NO

If yes, please describe the barrier: ______________________________________

If yes, is the air handling for the rooms separate from the air handling for the rest of the ward?

YES NO

iii) No information / other (please specify):

____

B.1 Infrastructure features for infection control available

B.1.a) Use of Ante room/area adjacent to patient *isolation room* for doffing PPE YES NO

If yes, please specify:

i) Are the “Clean” entrance and “dirty” exit separated? (2 doors) YES NO

ii) Is the entrance/exit via same pathway (door) YES NO

No information / other (please specify):

____

B.1.b) *Isolation unit* layout

Are the entrance and exits to the unit separated (2 doors/paths) YES NO

i) Do the staff enter/exit via same pathway/door YES NO

ii) No information / other (please specify):

____

B.1.c) Are all of the EVD isolation rooms negative pressure patient isolation rooms YES NO

If yes, please specify

i) Number of air changes per hour ______________ Quantity:__________

No information / other (please specify):

____

B.1.c) HEPA filtration YES NO

If yes, filtration of: intake air exhausted air both

No information / other (please specify):

____

B.1.d) On-site sterilization of medical waste YES NO

If yes, please specify

i) sterilization method: autoclave incinerator other

If yes, please specify

In the isolation unit itself In the hospital elsewhere

If no, process identified for Category A Infectious Substance disposal YES NO

No information / other (please specify):

____

B.2 Laboratory capabilities of isolation facility

B.2.a) Location of laboratory support (Check all that apply)

i) Located within the patient care room	YES	NO
ii) Located within the isolation unit	YES	NO
iii) Located within the same campus	YES	NO
iv) Located within the same city	YES	NO

No information / other (please specify):

____

B.3.b) Classification of laboratory support (Check all that apply)

i) Bedside Point of Care Testing	YES	NO
ii) Clinical laboratory	YES	NO
iii) Public Health laboratory	YES	NO

No information / other (please specify):

____

B.3.c) Biosafety designation of hospital laboratory

i) BSL-2

ii) BSL-3

iii) BSL-4

No information / other (please specify):

____

B.3.c) Biosafety designation of public health laboratory

i) BSL-2

ii) BSL-3

iii) BSL-4

No information / other (please specify):

____

C.1 Cost of establishing high-level isolation capability

C.1.a) Approximate total cost incurred to establish ETC capacity since June, 2014: $________________

Construction/facility modifications: $________________

PPE purchases: $________________

Staff training: $________________

Unit planning: $________________

Acquisition of lab testing equipment: $________________

Other unit equipment purchases (not PPE or lab equipment): $________________

D.1. Ebola treatment center consortium participation

D.1.a) Would your facility participate as a member in a consensus network of isolation units to establish infection control metrics, competencies, and peer review for high-level patient isolation centers? YES NO

If yes, please specify

Point of contact for consortium participation:

Name:____

E-mail:____

Survey sent to all Ebola Treatment Centers.

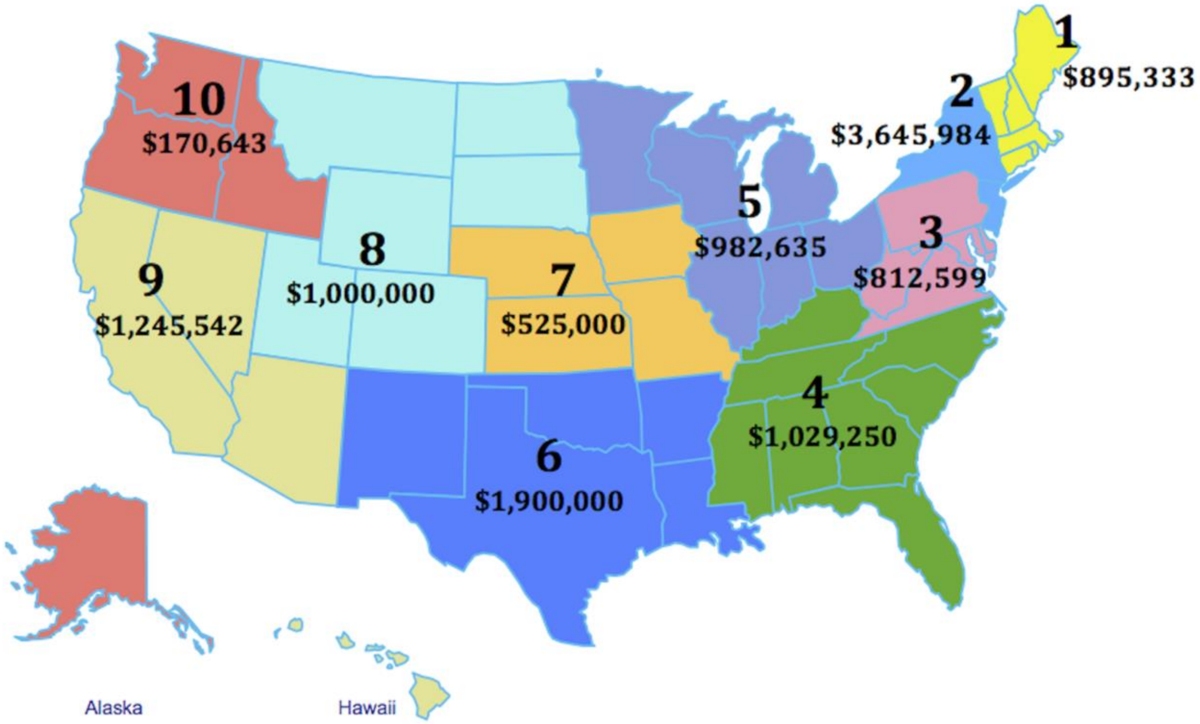

Technical Appendix Figure 1. Average total costs incurred in each of the 10 US Health and Human Services regions. Summarized data was collected through self-report by individual treatment centers through an electronically administered survey. [1]All Region 8 Ebola treatment centers provided estimates

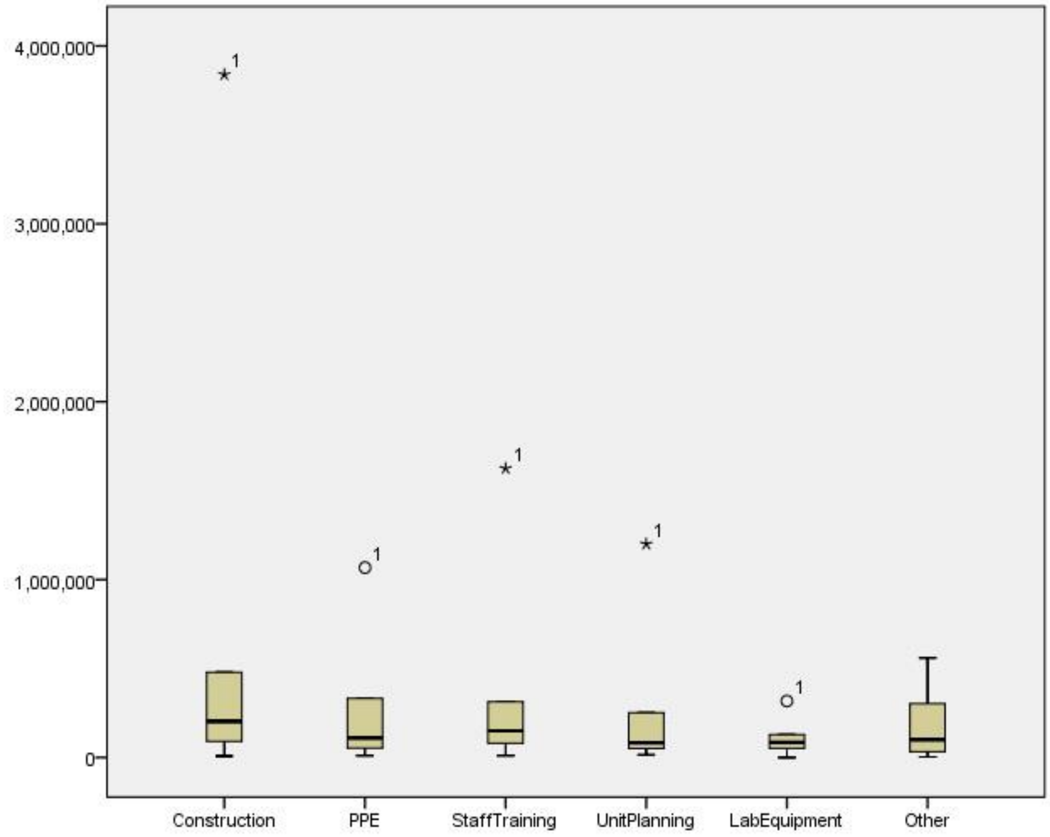

Technical Appendix Figure 2. Interquartile ranges of the distribution of costs of 45 Ebola treatment centers (US $). Data were collected through self-report by individual ETCs through an electronically-administered survey.
